# Explainable machine learning model for identifying key gut microbes and metabolites biomarkers associated with myasthenia gravis

**DOI:** 10.1016/j.csbj.2024.04.025

**Published:** 2024-04-10

**Authors:** Che-Cheng Chang, Tzu-Chi Liu, Chi-Jie Lu, Hou-Chang Chiu, Wei-Ning Lin

**Affiliations:** a PhD Program in Nutrition and Food Science, Fu Jen Catholic University, New Taipei City, Taiwan; b Department of Neurology, Fu Jen Catholic University Hospital, Fu Jen Catholic University, New Taipei City, Taiwan; c Graduate Institute of Biomedical and Pharmaceutical Science, Fu Jen Catholic University, New Taipei City, Taiwan; d Graduate Institute of Business Administration, Fu Jen Catholic University, New Taipei City, Taiwan; e Artificial Intelligence Development Center, Fu Jen Catholic University, New Taipei City, Taiwan; f Department of Information Management, Fu Jen Catholic University, New Taipei City, Taiwan; g School of Medicine, Fu Jen Catholic University, New Taipei City, Taiwan; h Department of Neurology, Taipei Medical University, Shuang-Ho Hospital, New Taipei City, Taiwan

**Keywords:** Myasthenia gravis, Gut microbiota, Metabolites, Machine learning, Artificial intelligence, Explainable

## Abstract

Diagnostic markers for myasthenia gravis (MG) are limited; thus, innovative approaches are required for supportive diagnosis and personalized care. Gut microbes are associated with MG pathogenesis; however, few studies have adopted machine learning (ML) to identify the associations among MG, gut microbiota, and metabolites. In this study, we developed an explainable ML model to predict biomarkers for MG diagnosis. We enrolled 19 MG patients and 10 non-MG individuals. Stool samples were collected and microbiome assessment was performed using 16S rRNA sequencing. Untargeted metabolic profiling was conducted to identify fecal amplicon significant variants (ASVs) and metabolites. We developed an explainable ML model in which the top ASVs and metabolites are combined to identify the best predictive performance. This model uses the SHapley Additive exPlanations method to generate both global and personalized explanations. Fecal microbe–metabolite composition differed significantly between groups. The key bacterial families were *Lachnospiraceae* and *Ruminococcaceae*, and the top three features were *Lachnospiraceae*, inosine, and methylhistidine. An ML model trained with the top 1 % ASVs and top 15 % metabolites combined outperformed all other models. Personalized explanations revealed different patterns of microbe–metabolite contributions in patients with MG. The integration of the microbiota-metabolite features and the development of an explainable ML framework can accurately identify MG and provide personalized explanations, revealing the associations between gut microbiota, metabolites, and MG. An online calculator employing this algorithm was developed that provides a streamlined interface for MG diagnosis screening and conducting personalized evaluations.

## Highlights

• The explanatory ML strategy proposed a novel tool to personalized explanations and detection of MG.
• The ML model trained with top-significance gut ASV-metabolite had best diagnostic accuracy for MG.
• SHAP demonstrated personalized different patterns of microbe–metabolite contributions across MG.
• The fecal microbe-metabolite composition significant differences between MG and those without.
• Key biomarkers for diagnosis of MG were identified as *Lachnospiraceae*, inosine, and methylhistidine.

## Graphical Abstract

**Figure:**
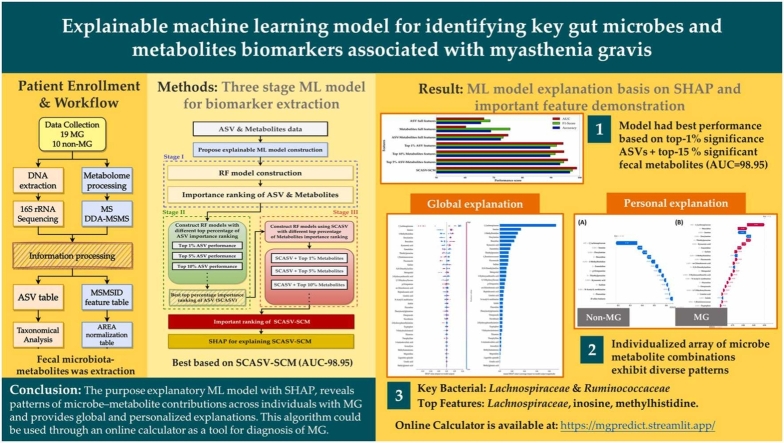

## 1 Introduction

Myasthenia gravis (MG) is a complex autoimmune disorder characterized by fluctuating muscle weakness resulting from autoantibodies targeting postsynaptic structures at the neuromuscular junction [1]. Regardless of the subtype, MG impairs multiple immunomodulatory and neuromuscular junction signaling cascades, thereby causing generalized or focal muscle fatigue [2]. The incidence of MG has gradually increased, with a current estimated incidence of 20 per 100,000 individuals [3]. This increase is attributable to enhanced diagnostic precision. Despite substantial improvements in disease diagnosis and management strategies, the precise etiology and mechanisms of MG remain unclear, and effective clinical tools for the accurate diagnosis and treatment of MG are limited [1]. Early identification of patients with MG may facilitate the use of advanced targeted immunotherapies. Therefore, the development of additional tools and biomarkers for predicting MG prognosis and personalizing management is imperative.

Changes in the composition of fecal microbiota profiles have been documented to impact the pathogenesis of several neurological disorders [4]. The progression of autoimmune diseases involves a complex interplay among various environmental, genetic, and microbial factors that contribute to immune dysregulation. Gut dysbiosis occurs in T-regulatory (Treg) cell–related autoimmune diseases such as inflammatory bowel disease and rheumatoid arthritis [5], [6]. Recent literatures have highlighted the connection between alterations in the fecal microbiota and immunomodulation of MG pathogenesis [7], [8]. Although the underlying mechanisms have yet to be elucidated, the immunomodulatory effects of gut microbiota are mostly realized through the T-helper 17 cell–Treg cell axis in MG, and dysbiosis is closely associated with an imbalanced cytokine profile and increase permeability of the gastrointestinal mucosal junction [9], [10]. MG is also accompanied by changes in fecal metabolites, which serve as functional aspects of the microbiome. functional indicator of the gut microbiome [11], [12]. Recent research has focused on MG-associated gut microbes. However, the association between gut microbiota and metabolism in MG remains unclear. Moreover, effective strategies and tools for analyzing gut microbes and metabolites for the diagnosis of MG are unavailable.

The expansion of microbiome research and advancements in sequencing technologies have increased the quantity and complexity of data. Consequently, more practical and advanced microbiome data analysis techniques require the use of machine learning (ML) to construct predictive frameworks that reveal complex interactions between microbial communities and host organisms [13]. Studies using such models have relied on taxonomically differentiated models, including those used for feature classification [14], [15], disease prediction [16], and microbial signature identification [17]. The increasing use of ML models in clinical settings has necessitated assessments of not only the predictive accuracy of such models but also the clarity of their recommendations [18]; specifically, model-provided predictions must be understandable, and this is often referred to as model “explainability.” The demand for interpretability has stimulated the development and application of interpretable AI/ML models for microbiome analysis [19]. Nevertheless, such mechanisms can be clarified through interpretable algorithms, which can lead to the elucidation of the complex interactions between gut microbiota and autoimmune disorders such as MG. Despite the aforementioned advantages of interpretable ML models, only a few microbiome studies have evaluated the potential of local explanation–based ML methods.

Although several studies have indicated that MG leads to altered gut microbial composition or dysbiosis, few studies have integrated gut microenvironment data to identify MG biomarkers. Accordingly, the present study addresses this research gap by integrating gut microbiota and metabolite data using ML to diagnose MG and predict its prognosis. Our study aimed to identify the features of human gut microbiota-metabolite associated with MG using a novel interpretable ML analytical framework based on an explainable ML scheme, the SHapley Additive exPlanations (SHAP) method, thus enabling it to guide personalized analysis and treatment plans. Metabolomic studies and gut microbiome profiling have revealed varying compositions in patients with MG. Our explainable ML scheme based on gut microbe–metabolite offers valuable insights into the diagnosis of MG, identifies the importance of individual features, clarifies MG-associated gut microbiota and metabolites, and guides personalized interventions in this intricate disorder.

## 2 Patients and methods

### 2.1 Patient enrollment

This study included patients who met the diagnostic criteria for MG (MG group) and individuals without MG (non-MG group). All diagnoses of MG were made by a neurologist. The inclusion criteria were (1) having MG signs and symptoms, such as muscle weakness and fatigue, and symptom fluctuation; (2) being seropositive for autoantibodies; (3) abnormal repetitive nerve stimulation or single-fiber electromyography; and (4) undergoing follow-up for ≥ 6 months from baseline. The exclusion criteria were (1) receiving aggressive immunomodulation therapy, including plasmapheresis, intravenous immunoglobulin, or rituximab, in the previous year or during the study inclusion period; (2) receiving antibiotics, probiotics in the previous 6 months; (3) having digestive disorders necessitating the use of a proton pump inhibitor or histamine-2 receptor antagonist, and disorders that related with change fecal microenvironments, such as inflammatory bowel disease in the previous year [20], [21]; or (4) requiring no immunosuppressant dose adjustment in the past one year. This study was approved by the Research Ethics Committee of Fu-Jen Catholic University Hospital (approval number: FJUH109042). Written informed consent was obtained from all participants.

### 2.2 Sample processing

**Fig. 1:**
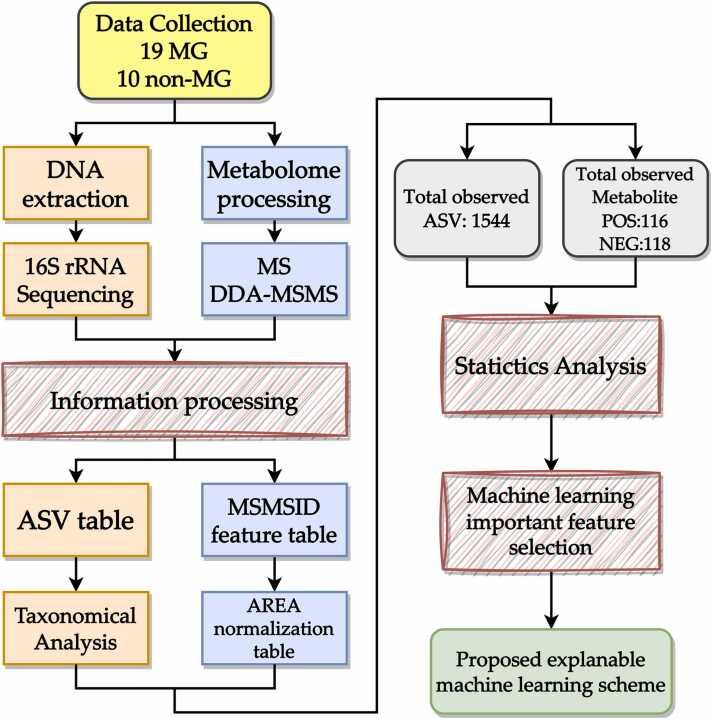
Flowchart of the study. The main steps in the study workflow are outlined as follows: microbial DNA extraction and rRNA sequencing; metabolite extraction and sample analysis; bioinformatic analysis; ASV table generation, data filtering, and taxonomic analysis; metabolite data preprocessing and annotation; and fecal metabolite feature table construction and area normalization. A total of 1544 ASVs were detected, and 116 positive and 118 negative metabolites were detected. A statistical analysis was conducted to compare the MG and non-MG groups in terms of gut microbiota and metabolite profiles. Additionally, we performed hyperparameter tuning and cross-validation processes for our random forest model–based feature selection framework to optimize model performance and identify the most important features for accurate predictions. Finally, to provide individual and global explanations for the importance of features, we developed an explainable machine learning scheme. ASV, amplicon sequence variant; MG, myasthenia gravis.

The comprehensive research process is illustrated in Fig. 1. The following steps were adopted to identify amplicon sequence variants (ASVs), assess metabolite functionality, perform taxonomic analysis, and develop an explainable ML scheme: gut microbiota and metabolite sampling, DNA extraction and sequencing, metabolite extraction, and data preprocessing and annotation (orange box, described in the following sections). Before data processing, we performed taxonomic analysis to identify significant microbiota–metabolite differences between the two groups. Metabolites were subjected to functional and taxonomic analyses using various databases. Furthermore, the correlations between the different metabolite profiles of the two groups were analyzed. Subsequently, an ML model based on the random forest (RF) algorithm was trained to predict MG. After the optimal hyperparameters were selected, interpretable ML models were used to produce the best predictive explanations.

### 2.3 Experiments

#### 2.3.1 Sample collection and processing

Fecal samples were collected and immediately stored at − 80 °C with quality control measures conducted at each stage.

#### 2.3.2 16S RNA sequencing and analysis

Microbial DNA process and sequencing were performed from our previous investigation [22]. Briefly, fecal DNA was extracted using the EasyPrep Stool Genomic DNA Kit (Biotools, New Taipei City, Taiwan). The V3–V4 regions of 16 S rRNA were amplified by PCR using specific primers. A polymerase chain reaction (PCR) utilized the KAPA HiFi HotStart ReadyMix kit (Roche) with genomic DNA as the template. The 16S Metagenomic Sequencing Library Preparation procedure from Illumina involved secondary PCR using the Nextera XT Index Kit and Illumina adapters. The library, composed of equal proportions of indexed PCR products, was sequenced on an Illumina MiSeq platform to produce 300‐bp paired-end reads.

#### 2.3.3 Metabolite extraction, sample preprocessing, and annotation

Untargeted metabolic profiling was performed using stool samples from the participants. After vortexing, homogenization, and sonication, the samples were incubated and centrifuged, and the supernatant was used for further experiments. Quality control involved mixing equal aliquots of supernatants, and 10 µL was injected into an ultra-high-performance liquid chromatography (UHPLC) system (Vanquish Horizon UHPLC System; Thermo Fisher Scientific, USA) coupled with a mass spectrometer (MS) (Orbitrap Elite; Thermo Fisher Scientific). Chromatography parameters included a column with specific dimensions, temperature, and binary mobile phase. Linear gradient elution was applied. To avoid carryover effects, a blank injection was performed after every sample injection. To normalize the peak areas, one quality control injection was performed after every five sample injections.

Positive-ion mode MS data were acquired using the default data-dependent acquisition method. Metabolite data preprocessing included conversion to mzML format, processed using the R (version 3.2) package XCMS, and generation of a data matrix. Normalization and quantification were based on the area under the receiver operating characteristic curve (AUC) values, with peaks annotated using an in-house MS2 database. The annotation threshold score was set at 0.5. Multivariate analysis involved outlier removal, missing data handling, and normalization considering total peak area [23], [24]. MS/MS identification matching scores were set at 0.5, and compounds were functionally and taxonomically annotated using the Kyoto Encyclopedia of Genes and Genomes and Human Metabolome databases.

#### 2.3.4 Statistical analysis

##### 2.3.4.1 16S rRNA sequencing analysis

For amplicon sequencing, we used paired-end raw reads and sorted our samples using their barcodes. Unnecessary sequences were removed using the QIIME2 cutadapt plugin [25]. Next, ASVs were identified using a denoising pipeline with the QIIME2 DADA2 plugin (version 2021.4), which involved several steps to clean the data and remove errors [26]. We then used QIIME2 to classify these sequences using the Silva database and compared ASVs with known sequences [27], [28]. Finally, statistical analysis was conducted utilizing Welch's *t*-test.

##### 2.3.4.2 Analysis of metabolite profiles

Multivariate analyses (MVA) of the metabolite profiles were conducted using principal component analysis (PCA). Additionally, orthogonal partial least squares discriminant analysis (OPLS-DA) was also employed for MVA. Different metabolites were filtered based on variable importance in projection (VIP) scores obtained from the OPLS model. The student *t*-test was utilized to compare between groups. VIP score ≥ 1 and *p* value < 0.05 was considered significant. For univariate analysis, metabolites were analyzed using volcano plots that met the following criteria: |log2fc| < 1 and *p* ≤ 0.05.

#### 2.3.5 Machine learning modeling

This study applied random forest (RF) to construct the ML methods. For hyperparameter tuning, we adopted the nested leave-one-out cross-validation approach (LOOCV) for each RF model. Performance was evaluated using the following metrics: sensitivity, specificity, accuracy, F1 score, and AUC. We conducted our experiments using Python (version 3.8.8) and a Jupyter Notebook (version 6.3.0) [29], [30]. LOOCV and hyperparameter tuning were performed using the Scikit-learn package API [31]. The SHAP method enabled us to extract global importance rankings for different groups and provide personalized explanations [32]. Finally, the extracted information was discussed in the context of this study.

## 3 Results

### 3.1 Clinical characteristics

The MG and non-MG groups consisted of 19 and 10 individuals, respectively. No antibiotics or probiotics were used by the participants during the enrollment period. Our cohort showed that 8 of 19 (42 %) patients had thymic pathology. The characteristics of the participants are summarized in Supplementary Table 1 and the characteristics of the thymoma and non-thymoma MG are summarized in Supplementary Table 2**.**

### 3.2 Gut Microbial Compositions in the MG and Non-MG Groups

From the samples, a total 1544 ASVs was identified. A rarefaction curve analysis and a species accumulation boxplot (Supplementary Fig. S1A) indicated that our sequencing data and sample sizes were adequate. An UpSet plot of the findings revealed some ASVs unique to subjects with and without MG (Supplementary Fig. S1B).

To identify noteworthy distinctions in the gut microbiomes between the two groups, we compared the groups in terms of the relative abundance of various microbes at several taxonomic levels (Supplementary Fig. 2), which revealed significant between-group differences. Representative examples of the top 10 most abundant bacteria at the different taxonomic levels detected in the two groups are presented in Supplementary Fig. 2A, B. Welch’s *t* test was utilized to investigate whether the MG group exhibited any significant reduction in the abundance of any taxonomic group compared with the non-MG group, as well as to identify potential species with significant between-group differences (*p* < 0.05). Subsequently, we generated a bar chart illustrating the intergroup species variation at the phylum, family, and genus levels (Supplementary Fig. 2C–E). Our findings suggest significant variations in the composition of fecal microbiomes among the different groups.

### 3.3 Fecal metabolite profiles in the MG and Non-MG groups

**Fig. 2:**
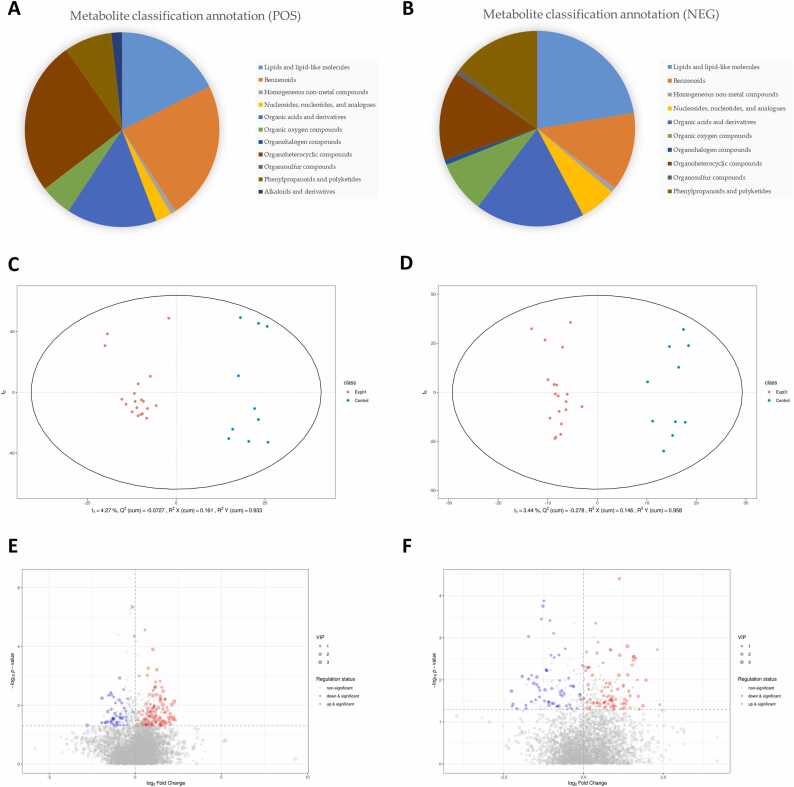
Proportions of various classes of fecal metabolites detected in all patients. Proportions of fecal metabolite classes in the positive (A) and negative (B) ion modes. Scatter plots of the OPLS-DA scores in the positive (C) and negative (D) ion modes. The x-axis presents the sample scores on the first principal component, and the y-axis presents the sample scores on the second principal component. R2Y represents the interpretation rate of the model, Q2Y indicates the predictive ability of the OPLS-DA model. Volcano plots of the VIP scores in the positive (E) and negative (F) ion modes. The horizontal axis presents the fold changes compared with the control group, displayed on a logarithmic scale (log2). The vertical axis presents *p* values obtained from Student’s *t* test, also displayed on a logarithmic scale (log10). The size of each data point corresponds to the VIP score calculated in the OPLS-DA model, with larger points indicating higher VIP scores. The color of the points indicates the results of metabolite screening: red for significantly increased metabolites, blue for significantly reduced metabolites, and gray for metabolites with no significant differences between the two groups. The numbers on the plots indicate the top 10 metabolites with the smallest *p* values and the top 10 metabolites with the largest and smallest log2 fold changes. OPLS-DA, orthogonal partial least squares discriminant analysis; VIP, variable importance in projection.

In this study, we examined the fecal metabolites in the two groups. After preprocessing, we observed that the positive-ion mode retained 8433 peaks, whereas the negative-ion mode retained 5310. Moreover, after data normalization, the positive-ion mode retained 6226 peaks, whereas the negative-ion mode retained 3913 peaks. The cutoff for the MS/MS identification matching score was set at 0.5. In cases where multiple features corresponded to the same substance, only the feature with the highest score was retained. The positive and negative datasets had 116 and 118 correspondents, respectively. The classification and annotation results for the 234 metabolites (identified from the fecal samples) were obtained from the Human Metabolome Database (Fig. 2A, B). Among these metabolites, lipids and lipid-like molecules constituted the most prominent group in the negative-ion mode. The organoheterocyclic compounds constituted the most prominent group in the positive-ion mode (Supplementary Figs. 3 and 4).

To determine the between-group differences in fecal metabolites, a supervised OPLS-DA model was used. This model explained most between-group differences (Fig. 2C, D). The corresponding volcano plots are presented in Fig. 2E and F. The significance of the between-group differences is shown in Supplementary Figure 5. Several differences in fecal metabolites were observed between the MG and non-MG groups.

### 3.4 Proposed explainable ML approach

**Fig. 3:**
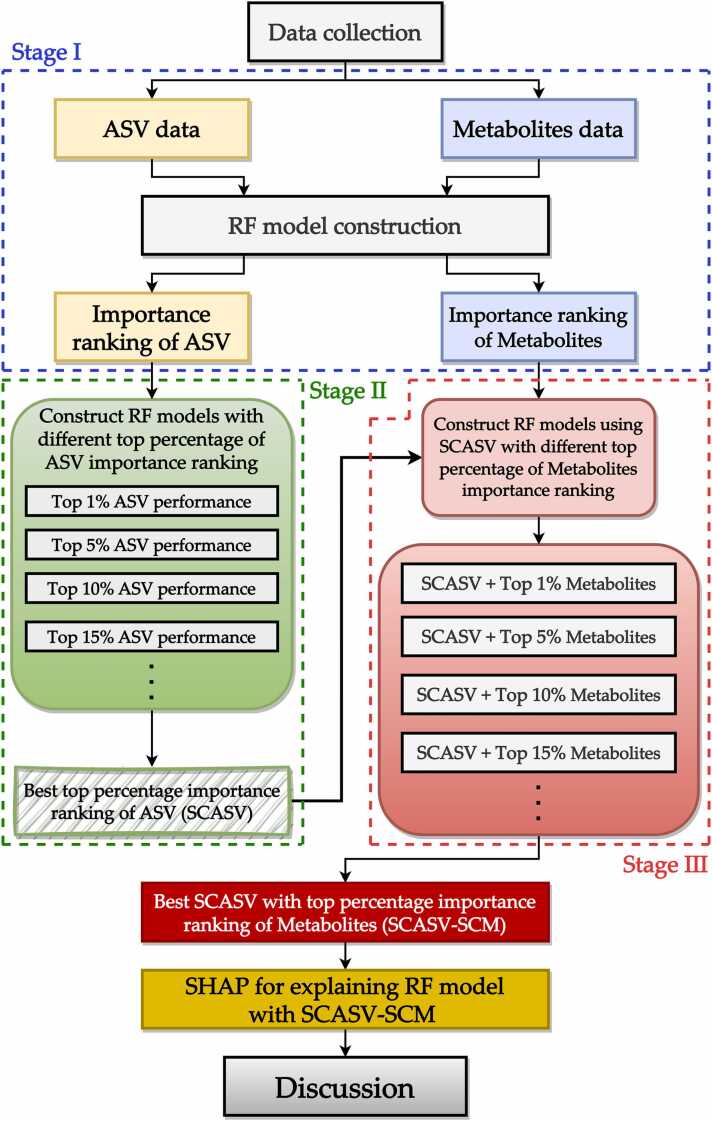
Proposed explainable machine learning scheme for selecting important features. Stage I: hyperparameter tuning and cross-validation of a random forest model. Stage II: evaluation of the performance of models constructed using different ASVs in the top percentiles. Stage III: combination of significantly contributing ASVs with metabolites in the top percentiles. Statistical analyses were conducted for feature ranking, and insights from the statistical tests were compared with those obtained using the Shapley Additive Explanations method. ASV, amplicon sequence variant.

We developed a three-stage ML model to select important features. Using this scheme, efficient predictive models were developed from the most relevant combinations of ASVs and metabolites. The proposed scheme primarily entails first identifying the best model trained with the top ASVs, and then identifying the best model trained with the significantly contributing ASVs and the most important metabolites. The SHAP method was employed to elucidate the final models. Fig. 3 shows the proposed explainable ML model for important feature selection. Detailed steps are described later sections (Sections 3.5 to 3.9). This model comprises three major stages. In stage I, ASV and metabolite features extracted from the collected data are divided into two datasets: one containing all ASV features, and the other containing all metabolite features. Next, RF models are constructed using these two datasets, and the features are ranked on the basis of their importance. In stage II, the optimal number of ASVs required to construct the best RF model is determined first. Given the substantial number of ASVs, the proposed scheme uses different top percentile thresholds (e.g., 1 %, 5 %, and 10;%) based on the previously identified feature importance rankings. For example, consider a scenario in which 1000 ASVs have been ranked. The top 1 % ASVs comprise the first 10 ASVs (1000 × 0.01 = 10), the top 5 % of ASVs comprise the first 50 ASVs (1000 × 0.05 = 50), and so on. RF models constructed using the top 1 % significance contribution ASVs (SCASVs) exhibited the best performance. In stage III, the objective is to combine the SCASVs with metabolites in the top percentiles. The combination associated with the best RF model performance was referred to as the SCASVs with top 15 % significantly contributing metabolite (SCMs) combination. Thus, the RF model constructed using the SCASVs and SCMs was used as the final predictive model, and the interpretation was facilitated based on the SHAP method. Finally, the model and SCASV/SCM combination was comprehensively interpreted.

### 3.5 ML model trained with ASV and/or metabolite data can predict MG

To evaluate the predictive performance of the models trained using gut microbiota and/or metabolite features, we used the ASVs and metabolite features to train supervised ML models, specifically RF models, to differentiate individuals with MG from those without MG (Fig. 3, Stage I). Table 1 presents the metrics for the performance of the models in classifying individuals with or without MG based on different datasets. Models trained using a combination of ASVs and metabolites exhibited the highest predictive performance (AUC, ∼0.75), followed by those trained using only ASV data (AUC, ∼0.666) and those trained using only metabolite data (AUC, ∼0.603). Thus, combining the gut microbiota and metabolomic data can improve the predictive ability of ML models.

**Table 1:** Metrics of model performance in classifying individuals with or without MG on the basis of different data sets.

Data	Accuracy	F1-score	Sensitivity	Specificity	AUC
**ASV data**	65.52	68.75	57.89	80.00	66.6
**Metabolite data**	68.97	75.68	73.68	60.00	60.3
**ASV–metabolite data**	72.41	73.33	57.89	100	75.0

ASV, amplicon sequence variant; AUC, area under the characteristic curve.

### 3.6 Random forest models trained with ASV and/or metabolite features can diagnose MG

To investigate whether the use of gut microbiota and/or metabolite features can improve the accuracy and precision of diagnostic classification, we trained the RF models with features selected from various datasets (Fig. 3, Stage II). Specifically, various top-percentile features were selected from the ASV, metabolite, and combined datasets. The top-percentile features were selected based on the importance rankings directly calculated by the models. To evaluate whether the model trained with the selected top-percentile features could effectively classify individuals with or without MG, RF modeling was performed as previously described. Specifically, the models were trained using the top 1 %, 5 %, 15 %, and 20 % of the features from each dataset. As presented in Table 2, the RF model trained with the top 1 % ASV features had higher AUC values (∼ 0.94) than those trained without feature selection (Table 2 and Table 1). The 1 % ASV subset comprised the significantly contributing ASVs (SCASVs). The incorporation of the top 10 % metabolite features led to higher AUC values than those obtained under scenarios without feature selection. Thus, selection of the top features can enhance the diagnostic ability of ML models, regardless of the dataset type.

**Table 2:** Metrics of model performance in classifying individuals with or without MG on the basis of the top ASV and/or metabolite features.

Data sources	Top features	Accuracy	F1-score	Sensitivity	Specificity	AUC
**ASV data**	**Top 1 %**	89.66	91.89	89.47	90.00	94.21
	**Top 5 %**	89.66	91.89	89.47	90.00	92.11
	**Top 10 %**	89.66	91.89	89.47	90.00	86.84
	**Top 15 %**	75.86	77.42	63.16	100	85.53
	**Top 20 %**	72.41	75.00	63.16	90.00	77.37
**Metabolites**	**Top 1 %**	93.10	94.74	94.74	90.00	94.21
	**Top 5 %**	89.66	92.31	94.74	80.00	92.63
	**Top 10 %**	89.66	91.43	84.21	100	94.47
	**Top 15 %**	89.66	91.89	89.47	90.00	90.53
	**Top 20 %**	82.76	84.85	73.68	100	92.11

ASV, amplicon sequence variant; AUC, area under the characteristic curve.

### 3.7 ML models trained with SCASVs/SCMs can precisely identify MG markers

**Fig. 4:**
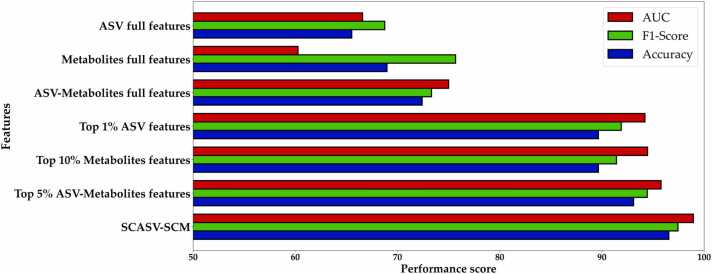
Assessment of different feature selection data sets using a random forest model. Bar plots comparing the predictive power were derived from the test data set using the GA-optimized classifier with other predictors by (1) area under the receiver operating characteristics curve (AUC) (Red bar), (2) F1-score (Green bar), and (3) accuracy (Blue bar). ASV, amplicon significant variants; SCASV, significance contribution ASVs; SCV, significantly contributing metabolite.

**Fig. 5:**
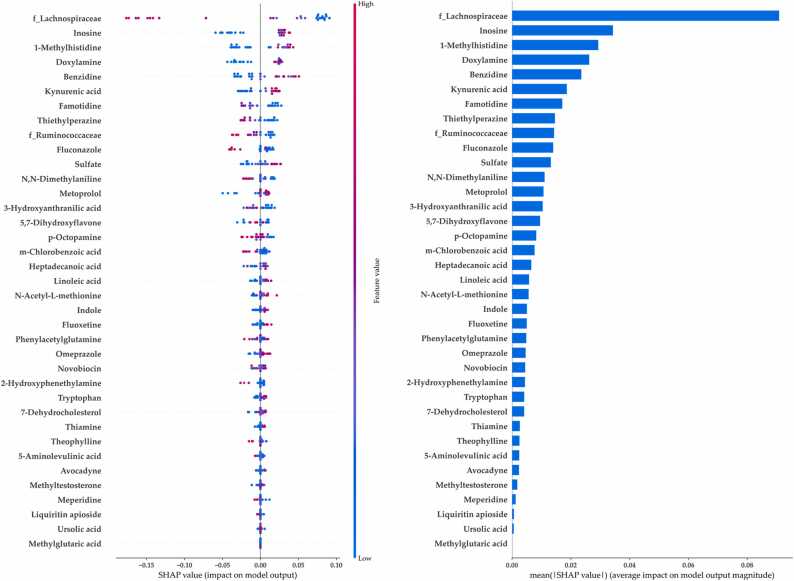
SHAP summary plot (left) and bar plot (right) for the top features based on the random forest model. Higher SHAP values indicate higher likelihoods of having myasthenia gravis. The summary plot (left) shows the effect directions as well as specific bacteria and metabolites exhibiting differential effects on each individual. In this plot, each patient is represented by a dot in each variable, the dots are colored according to the feature values for each participant, red dots represent increased abundance, and the blue dots indicate reduced abundance. The x-axis represents the feature values, and the y-axis represents the SHAP values; The red-dot clusters indicate increased abundances of taxa corresponding to presented on the y-axis. This plot shows changes in the importance of a feature with its value; for example, in patients with MG, the relative abundance of the family *Lachnospiraceae* decreased, but the proportion of inosine increased. Accordingly, SHAP values exceeding 0 were considered to indicate an increased likelihood of MG diagnosis. The bar chart of global feature importance (right) was constructed by the mean SHAP value of each feature. This chart shows the average effect of each feature on the model output. In contrast to the summary plot, the bar chart does not show the effect directions or bacteria exhibiting low global feature importance but high influence. MG, myasthenia gravis; SHAP, Shapley Additive exPlanations.

To further improve the diagnostic ability of the ML models and reduce the dimensionality of the ASV feature space simultaneously, we assessed the performance of the ML models by pairing SCASVs with varying top-percentile metabolites (Fig. 3, Stage III). RF models were trained using combinations of SCASVs with the top 1 %, 5 %, 15 %, and 20 % metabolites. Notably, we observed that the combination of SCASVs with the top 15 % metabolites (SCMs) resulted in improved model performance (AUC, ∼0.99; sensitivity, ∼1.00) compared with the other combinations (Table 3). Furthermore, we compared the model performance levels achieved using ASV data, metabolite data, ASV–metabolite data, top 1% ASV data, top 10 % metabolite data, top 5 % ASV–metabolite data, and SCASV/SCM. Model performance was evaluated using accuracy, F1 score, and AUC metrics (Fig. 5). The SCASV/SCM dataset showed better model performance than the other datasets (Table 4 and Fig. 4). Therefore, the RF model trained with SCASVs (top 1 %) combined with SCMs (top 15 %) exhibited strong diagnostic classification power (Fig. 4). A total of 48 features—13 ASVs and 35 metabolites—were extracted for the ML models. Supplementary Tables 3–5 present detailed information on the selected important features.

**Table 3:** Metrics of model performance in classifying individuals with or without MG on the basis of the top 1 % ASV and top metabolite features.

SCASV	Top % metabolites	Accuracy	F1-score	Sensitivity	Specificity	AUC
**Top 1%**	**Top 1 %**	96.55	97.30	94.74	100	98.42
**Top 1%**	**Top 5 %**	96.55	97.30	94.74	100	97.11
**Top 1%**	**Top 10 %**	96.55	97.30	94.74	100	96.32
**Top 1%**	**Top 15 %**	96.55	97.44	100	90.00	98.95
**Top 1%**	**Top 20 %**	93.10	94.44	89.47	100	97.89

ASV, amplicon sequence variant; AUC, area under the characteristic curve.

**Table 4:** Numbers of ASV and metabolite features for models constructed using the top 1 % ASVs and different top-percentile thresholds of metabolites.

TSCASV	Top % Metabolites	Number of ASV features	Number of metabolites features	Total features	AUC
**Top 1%**	**Top 1 %**	13	2	15	98.42
**Top 1%**	**Top 5 %**	13	11	24	97.11
**Top 1%**	**Top 10 %**	13	23	36	96.32
**Top 1%**	**Top 15 %**	13	35	48	98.95
**Top 1%**	**Top 20 %**	13	46	59	97.89
**Top 1%**	**Top 30 %**	13	70	83	93.95
**Top 1%**	**Top 40 %**	13	93	106	92.63

ASV, amplicon sequence variant; AUC, area under the characteristic curve.

### 3.8 Explainable ML models can identify key gut microbes and metabolites associated with MG

To identify the most influential features in our predictive model, we conducted SHAP analysis. Thus, we visualized and ranked the SCASV and SCM features according to their importance. The SHAP values of all the individuals were used to create a summary plot (Fig. 5, left). SHAP analysis was conducted using the aforementioned 48 ASV–metabolite features (Supplementary Tables 3 and 4), including the bacterial families *Lachnospiraceae* and *Ruminococcaceae*. The summary plot illustrates the relationship between high and low feature values and the SHAP values within the dataset. This plot illustrates how different features affect the diagnosis of MG. In patients with MG, the family *Lachnospiraceae* decreased, but inosine increased. SHAP values > 0 suggested a higher risk of MG. According to the predictive model, a higher SHAP value indicated a higher likelihood of MG diagnosis. The top three key features for the diagnosis of MG were *Lachnospiraceae*, inosine, and methylhistidine. The SHAP dependence bar plot presented in Fig. 5 (right) can be used to understand the influence of a single feature on the output of the predictive models.

### 3.9 Personalized explanations reveal different sets of influential bacteria in patients with MG

**Fig. 6:**
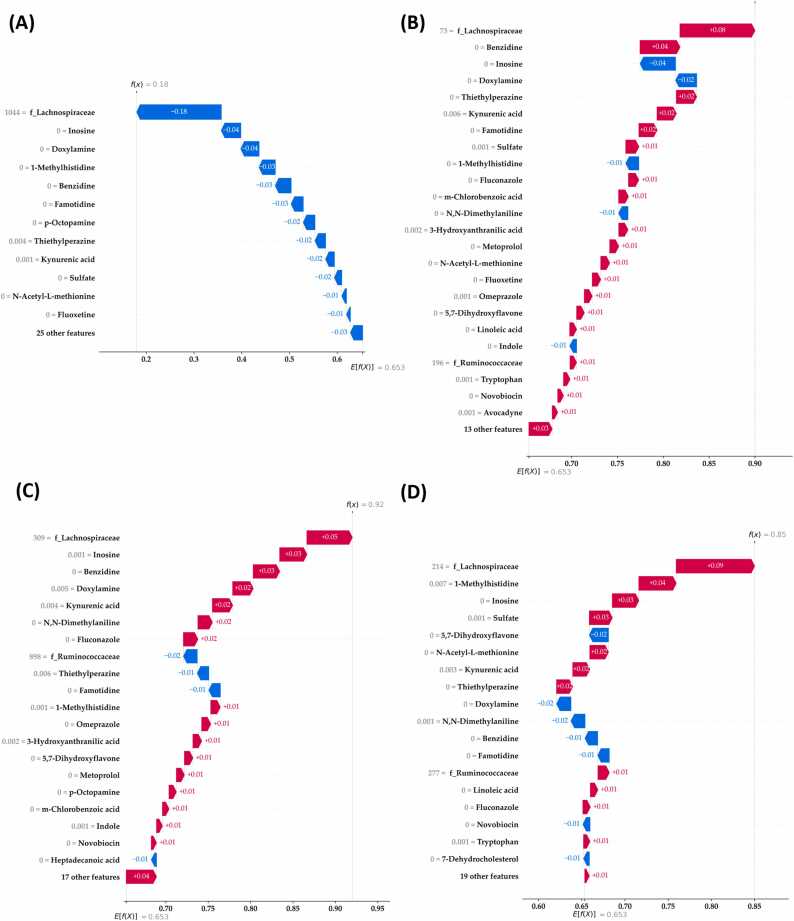
Examples of four individuals’ MG prediction results. Waterfall plots of personalized local explanations for (A) one individual without MG and (B–D) three individuals with MG. At the bottom of each plot, we can see the base value (0.653), which represents the expected value of MG (mean model output across the training data). The sum of all SHAP values for each local explanation equals the predicted likelihood of MG diagnosis (SHAP-based personalized prediction). MG, myasthenia gravis; SHAP, Shapley Additive exPlanations.

To generate personalized explanations using the SHAP method, we selected four participants: three with MG and one without MG (Fig. 6). Each explanation involved SHAP values representing the contribution of each feature to the prediction made by the classifier. We created waterfall plots, with the SHAP values in each plot arranged vertically in descending order (from largest to smallest); thus, the most influential gut microbiota–metabolite combination for a given individual was listed at the top. For each personalized explanation, the sum of all relevant SHAP values was equal to the classifier output (predicted likelihood of MG diagnosis). For example, the sum of all SHAP values for the first patient with MG was 0.9, representing the classifier-predicted likelihood of MG diagnosis. This property is called local accuracy [11]. In individuals without MG (Fig. 6A) and those with MG (Fig. 6B), the family *Lachnospiraceae* made the largest contribution to the classifier’s prediction, followed by the inosine proportion. These local explanations revealed different patterns of microbe–metabolite combinations in patients with MG. This result could not be achieved using any global ML model (including RF models). These explanations suggest that in the patients whose examples are presented in Fig. 6B–D, *Lachnospiraceae* was ranked as the most important feature in the overall SHAP analysis, but inosine did not have a relatively high or positive contribution to the prediction in different individuals. Based on our validated database, our ML predictive model for MG can be utilized as an online calculator by inputting the required data. Available at https://mgpredict.streamlit.app/ (see Supplementary Appendix for details).

## 4 Discussion

To our knowledge, this study is the first to demonstrate the potential of explainable ML models for predicting the likelihood of MG diagnosis and explaining the predictions in terms of variations in gut microbial composition across individuals. We conducted integrated metabolomic and metagenomic analyses to identify MG-associated multiomics features and developed an explainable ML scheme for identifying MG biomarkers. This study determined that RF models trained using the top-percentile features yielded predictions superior to those obtained from direct analyses conducted using the entire dataset. The ML models trained with either ASVs or metabolite features yielded relatively unsatisfactory predictive performance (AUC, ∼0.60–0.66); however, when we combined the two datasets, the model’s predictive performance increased (AUC, ∼0.75). The families *Lachnospiraceae* and *Ruminococcaceae* and the phylum Firmicutes, which are the top 1 % SCASVs, were the key taxa detected in this study. We identified distinct microbial signatures and combined these features with the top 15 % SCMs to train effective ML models to differentiate between MG and non-MG groups. Finally, 13 ASVs and 35 metabolite features were selected using the SHAP method. The feature importance ranking revealed that the three most important features were *Lachnospiraceae*, inosine, and methylhistidine. We also evaluated SHAP-based personalized explanations for model predictions regarding MG diagnosis and biomarkers and identified different patterns of microbe–metabolite contributions to the likelihood of MG diagnosis across patients. Finally, we developed an online calculator that allows easy analysis of the human gut microbiome to predict MG based on the current cohort. Our simple framework should enable clinicians to easily utilize samples of the gut microbiome as a tool to support the diagnostic screening of MG and personalized analysis. Our proposed ML model revealed hidden local patterns that are generally not identified by the current global explanation models.

To reduce the dimensionality of the feature space and enhance the predictive performance of the ML scheme, we computed importance scores for the ASVs and identified the top 1 % ASVs associated with the highest predictive performance. The top 1 % ASV features—representing the most valuable ASVs identified from the samples collected from the MG and non-MG groups—were used for modeling, which increased the AUC value to approximately 0.94. ASVs can help differentiate between bacteria based on their DNA sequence similarity, and these features are more informative than taxonomic classes. Furthermore, fecal metabolomics can shed light on microbial activity and host–microbe interactions. Thus, we combined the 13 SCASVs with the top-percentile metabolites to identify the combination associated with the best predictive performance. Additional tests were conducted to determine whether using the top features to train the ML models could efficiently improve their predictive ability. The ML model trained with the top 1 % ASVs (n = 13) and top 15 % metabolites (n = 35) achieved the highest diagnostic accuracy. The AUC obtained in the final test was approximately 0.98. These SCASV and SCM features can serve as biomarkers of MG. Our feature selection results revealed that a small set of ASVs and metabolites can reduce model variability and enhance diagnostic accuracy. Nevertheless, future studies should further explore the selected SCASVs and SCMs to determine their potential implications in MG.

The use of microbiome data is challenging because they are characterized by high dimensionality, underdetermined features, and overdispersion. These unique characteristics hinder the effectiveness of conventional models for obtaining satisfactory analysis results [33]. ML models have been proven to be effective for analyzing complex datasets [19], [34], [35]. Additionally, advancements in ML-based tools used for the analysis of microbe–metabolite data have played vital roles in the optimization of best practices [36]. Conventional ML models, including support vector machine, k-nearest neighbor, and RF models, are commonly used to predict the characteristics of hosts and microbes [19], [37]. However, certain methodological challenges remain to be addressed, including the heterogeneity of microbiome data and the multitude of factors that influence sample properties. The RF algorithm, which is a widely used ensemble classification technique, typically employs decision trees as base classifiers and combines their outputs iteratively [38]. However, global ML approaches can only represent trends within the entire population and cannot analyze individual variations [39]. Consequently, their application in personalized medicine is limited.

Clinicians using traditional ML models often encounter difficulties navigating through different features and interpreting how such features affect the target variable. To avoid this, the proposed scheme uses the SHAP method. This method explains model predictions by extending the concept of Shapley values from cooperative game theory [40]. The mechanism of action of SHAP is straightforward. It assigns a contribution value to each predictive feature by comparing the prediction made with a given feature with that made without it [32], and the difference between the two predictions represents the contribution of that particular feature [32]. When assigning contribution values, the SHAP method simultaneously considers all possible feature combinations. The contribution of each feature to a predicted outcome may also vary, which may affect the outcome positively or negatively. Overall, the SHAP method provides insights into the extraction of genetic features that influence predicted outcomes. With the increasing popularity of SHAP, clinical studies have increasingly used this method for explaining model features [41], [42]. Our analytical framework effectively integrates data from different dimensions and can yield accurate results. The model explanations not only improve clinicians’ understanding of the associations between microbiome composition, metabolites, and phenotypes but also increase their confidence in the predictive ability of their models. Furthermore, SHAP-based personalized explanations can help identify different patterns of microbe–metabolite contributions in patients with MG. Hence, our findings demonstrate the potential of microbiome-based explainable ML models for predicting MG and facilitating personalized care of patients with this condition.

The use of 16 S metagenomic and metabolite data for MG prediction is not a novel approach, and most studies have applied conventional microbiome analysis methods. Moris et al. described gut dysbiosis in MG and reported that the abundance of Bifidobacterium spp. was increased in these patients [43]. Furthermore, a cohort study demonstrated that the gut microbiome and fecal metabolome vary across MG subtypes [11]. Another cohort study reported that MG not only alters the composition of the gut microbiota but also reduces the content of short-chain fatty acids (SCFAs) in fecal samples [12]. Zheng et al. revealed an association between MG and clinical variables and changes in the gut microbiome–metabolome [7] and observed significant correlations between bacterial operational taxonomic units (OTUs) and various metabolite biomarkers, with some OTUs being associated with the severity of MG. In contrast to that study, which utilized overall OTUs as the foundation for prediction, our study identified key features based on the top 1 % of significant ASVs. As indicated in previous studies, the use of top-ranking OTUs/ASVs can provide rich feature information that contributes to the enhancement of ML models [44]. In addition, research has revealed that even when several OTUs belong to the same bacterial species, different outcomes (increase or decrease) may occur [7]. Our explainable ML model can provide explanations for this condition, highlighting variations in the effects within the same bacterial strain across different individuals. In contrast to the aforementioned studies, our study used the proposed ML scheme, which achieved a promising AUC (∼0.98), indicating that the selected major influential features can provide reliable global and individualized results.

This study revealed that taxa belonging to the order Clostridiales and the family *Lachnospiraceae* were enriched in the non-MG group relative to the MG group. MG has been reported to alter microbes belonging to Clostridiales and *Lachnospiraceae* [8], [12]. A possible mechanism underlying the association between MG and gut microbiota is the production of SCFAs, which promote the differentiation of Tregs through various pathways [45], [46]. In the present study, inosine, a key metabolite derived from adenosine, was enriched in the MG group. This metabolite was associated with strong predictive power for MG. Inosine serves as an intracellular purine nucleoside [47] and an adenosine receptor agonist and is involved in the regulation of numerous physiological and pathophysiological processes [47]. Certain gut bacteria produce inosines. Mager et al. demonstrated that inosine derived from gut microbiota enhanced the efficacy of immune checkpoint inhibitors by activating antitumor T cells [48]. Inosine improves intestinal function through peroxisome proliferator–activated receptor-γ signaling, which is involved in the regulation of immune cell proliferation and differentiation [49]. This signaling cascade induces Treg differentiation [50]. Collectively, our findings emphasize the significance of *Lachnospiraceae* and inosine in maintaining a fine-tuned equilibrium in the immune response. Future studies should investigate the function of each taxon and its relationship with key metabolites.

Our study had several limitations. First, we used a cross-sectional design to explore MG-associated gut microbes and metabolites; however, we could not establish a causal relationship. Further research is required to identify these relationships. Secondly, the sample size was insufficient to demonstrate the power of our results. Although not perfect, we employed LOOCV to enhance our model’s predictive ability with a core set of potential biomarkers for MG and tried to develop a supportive tool for the diagnosis of MG. However, it is essential to validate our findings in a large, multicenter patient cohort and undergo external validation to improve the robustness of our conclusions in future trials. Third, medication status varied across the recruited patients with MG, potentially affecting the composition of their gut microbiota. Thus, future studies should adjust for medication-related variations. Finally, we did not document the dietary habits in the cohort. There is limited evidence from studies demonstrating the association between dietary habits, gut microbiota, and disease. Most microbiome analyses related to specific diseases did not collect dietary data, such as Food Frequency Questionnaires, 24 h recalls, or food diaries. In future research, we will incorporate the analysis of patients’ dietary habits to gain a better understanding of this relationship. A multi-region, multicenter prospective longitudinal study should be conducted because our cross-sectional study had limitations in predicting the likelihood of MG in healthy individuals due to its inherent nature. The pathophysiology of MG associated with specific bacterial taxa should be elucidated, blood data analyzed, and interpretable ML models explored to provide comprehensive result into the micro-environment on MG. In addition, we aim to development of a user-friendly web server based on our model and an online calculator. The online calculator will undergo continuous improvement by incorporating additional annotation systems and analysis modules that can enhance the applicability of the model across diverse populations and facilitate regular database updates.

## 5 Conclusion

Our explainable ML scheme can accurately screen patients with MG and provide personalized explanations to enable assessors to understand the associations between gut microbiota, metabolites, and MG. Our model can successfully identify patient-specific gut microbial features associated with MG. Thus, features with a high degree of influence either directly contribute to MG or serve as indirect indicators. The proposed ML scheme offers key insights into the complex interactions between microbes and metabolites, and can thus serve as a valuable tool for the early detection and monitoring of MG. Assisting disease screening with our ML model incorporated into an online calculator may facilitate more precise management of MG.

## Funding

This work is supported by grants from the Fu Jen Catholic University Hospital (PL-202108033-V to S.F.H.) and Fu Jen Catholic University Hospital (109-FJUH-06).

## CRediT authorship contribution statement

Che-Cheng Chang was involved in the data curation, methodology, formal analysis, interpretation data and writing-original draft. Wei-Ning Lin was involved in the study design, methodology, supervision, and writing—review and editing of the paper. Hou-Chang Chiu was responsible for data curation and methodology; Tzu-Chi Liu and Chi-Jie Lu conducted the software, visualization, analyzed and interpretation of the data. All authors have read and agreed to the published version of the manuscript.

## Declaration of Competing Interest

The authors declare that the research was conducted in the absence of any commercial or financial relationships that could be construed as a potential conflict of interest.

## Data Availability

The data that support the findings of this study are available from the authors can be found below: NCBI – https://doi.org/10.5061/dryad.73n5tb32m.

## References

[1] Gilhus N.E., Verschuuren J.J. (2015). Myasthenia gravis: subgroup classification and therapeutic strategies. Lancet Neurol.

[2] Gilhus N.E. (2016). Myasthenia gravis. N Engl J Med.

[3] Punga A.R. (2022). Epidemiology, diagnostics, and biomarkers of autoimmune neuromuscular junction disorders. Lancet Neurol.

[4] Jangi S. (2016). Alterations of the human gut microbiome in multiple sclerosis. Nat Commun.

[5] Zhou Y. (2018). Gut microbiota offers universal biomarkers across ethnicity in inflammatory bowel disease diagnosis and infliximab response prediction. mSystems.

[6] de Groot P.F. (2017). Distinct fecal and oral microbiota composition in human type 1 diabetes, an observational study. PLoS One.

[7] Zheng P. (2019). Perturbed microbial ecology in myasthenia gravis: evidence from the gut microbiome and fecal metabolome. Adv Sci (Weinh).

[8] Thye A.Y. (2022). Exploring the gut microbiome in myasthenia gravis. Nutrients.

[9] Chen P., Tang X. (2021). Gut microbiota as regulators of Th17/Treg balance in patients with myasthenia gravis. Front Immunol.

[10] Rinaldi E. (2018). Gut microbiota and probiotics: novel immune system modulators in myasthenia gravis. Ann N Y Acad Sci.

[11] Tan X. (2020). Differential gut microbiota and fecal metabolites related with the clinical subtypes of myasthenia gravis. Front Microbiol.

[12] Qiu D. (2018). Altered gut microbiota in myasthenia gravis. Front Microbiol.

[13] Soueidan H., Nikolski M. (2017). Machine learning for metagenomics: methods and tools. Metagenomics.

[14] Vervier K. (2016). Large-scale machine learning for metagenomics sequence classification. Bioinformatics.

[15] Werner J.J. (2012). Impact of training sets on classification of high-throughput bacterial 16s rRNA gene surveys. Isme J.

[16] Thomas A.M. (2019). Metagenomic analysis of colorectal cancer datasets identifies cross-cohort microbial diagnostic signatures and a link with choline degradation. Nat Med.

[17] Yachida S. (2019). Metagenomic and metabolomic analyses reveal distinct stage-specific phenotypes of the gut microbiota in colorectal cancer. Nat Med.

[18] Murdoch W.J. (2019). Definitions, methods, and applications in interpretable machine learning. Proc Natl Acad Sci.

[19] Li P. (2022). Machine learning for data integration in human gut microbiome. Micro Cell Fact.

[20] Kiecka A., Szczepanik M. (2023). Proton pump inhibitor-induced gut dysbiosis and immunomodulation: current knowledge and potential restoration by probiotics. Pharm Rep.

[21] Zhu J. (2023). Compared to histamine-2 receptor antagonist, proton pump inhibitor induces stronger oral-to-gut microbial transmission and gut microbiome alterations: a randomised controlled trial. Gut.

[22] Chang C.C. (2023). Machine learning strategy for identifying altered gut microbiomes for diagnostic screening in myasthenia gravis. Front Microbiol.

[23] Nagler M. (2018). Eco-metabolomics and metabolic modeling: making the leap from model systems in the lab to native populations in the field. Front Plant Sci.

[24] Li B. (2017). NOREVA: normalization and evaluation of MS-based metabolomics data. Nucleic Acids Res.

[25] Martin M. (2011). Cutadapt removes adapter sequences from highthroughput sequencing reads. EMBnet J.

[26] Callahan B.J. (2016). DADA2: High-resolution sample inference from Illumina amplicon data. Nat Methods.

[27] Bokulich, N., et al., Optimizing taxonomic classification of marker-gene amplicon sequences with QIIME 2′s q2-feature-classifier plugin. Microbiome 6, 1–17. *doi: 10.1186*. 2018, S40168–018-0470-Z/TABLES/3. 10.1186/s40168-018-0470-z PMC5956843 29773078

[28] Katoh K., Standley D.M. (2013). MAFFT multiple sequence alignment software version 7: improvements in performance and usability. Mol Biol Evol.

[29] Van Rossum G., Drake F.L. (2009).

[30] T. Kluyver et al. Jupyter Notebooks - a publishing format for reproducible computational workflows in International Conference on Electronic 2016 Publishing,.

[31] Pedregosa F. (2011). Scikit-learn: machine learning in python. J Mach Learn Res.

[32] Lundberg S.M., Lee S.-I. (2017). A unified approach to interpreting model predictions. Adv Neural Inf Process Syst.

[33] Xia Y., Sun J. (2017). Hypothesis testing and statistical analysis of microbiome. Genes Dis.

[34] Marcos-Zambrano L.J. (2021). Applications of machine learning in human microbiome studies: a review on feature selection, biomarker identification, disease prediction and treatment. Front Microbiol.

[35] Hernández Medina R. (2022). Machine learning and deep learning applications in microbiome research. ISME Commun.

[36] Moreno-Indias I. (2022). Editorial: microbiome and machine learning. Front Microbiol.

[37] Zhou Y.H., Gallins P. (2019). A review and tutorial of machine learning methods for microbiome host trait prediction. Front Genet.

[38] E. Goel E. Abhilasha, Random Forest: A Review. 2017.

[39] Rudin C. (2022). Interpretable machine learning: fundamental principles and 10 grand challenges. Stat Surv.

[40] Lundberg S.M., Lee S.-I. (2017). A unified approach to interpreting model predictions. ArXiv.

[41] Sun J. (2023). Application of SHAP for explainable machine learning on age-based subgrouping mammography questionnaire data for positive mammography prediction and risk factor identification. Healthcare.

[42] Ren H. (2023). Predicting acute onset of heart failure complicating acute coronary syndrome: an explainable machine learning approach. Curr Probl Cardiol.

[43] Moris G. (2018). Fecal microbiota profile in a group of myasthenia gravis patients. Sci Rep.

[44] Aryal S. (2020). Machine learning strategy for gut microbiome-based diagnostic screening of cardiovascular disease. Hypertension.

[45] Atarashi K. (2011). Induction of colonic regulatory T cells by indigenous Clostridium species. Science.

[46] Furusawa Y., Obata Y., Hase K. (2015). Commensal microbiota regulates T cell fate decision in the gut. Semin Immunopathol.

[47] Welihinda A.A. (2016). The adenosine metabolite inosine is a functional agonist of the adenosine A2A receptor with a unique signaling bias. Cell Signal.

[48] Mager L.F. (2020). Microbiome-derived inosine modulates response to checkpoint inhibitor immunotherapy. Science.

[49] Couvigny B. (2015). Commensal Streptococcus salivarius modulates PPARγ transcriptional activity in human intestinal epithelial cells. PLoS One.

[50] Nettleford S.K., Prabhu K.S. (2018). Selenium and selenoproteins in gut inflammation-a review. Antioxidants.

