# Supplementary material for: Explainable machine learning model for identifying key gut microbes and metabolites biomarkers associated with myasthenia gravis
Source: Comput Struct Biotechnol J. 2024 Apr 10;23:1572–83. doi: 10.1016/j.csbj.2024.04.025 (PMC11035017; doi:10.1016/j.csbj.2024.04.025)
Supplement: Supplementary file 1 — Supplementary material. [file mmc1.docx]

Supplementary Material

Explainable machine learning model for identifying key gut microbes and metabolites biomarkers associated with myasthenia gravis

Che-Cheng Chang, Tzu-Chi Liu, Chi-Jie Lu, Hou-Chang Chiu and Wei-Ning Lin

*** Correspondence:** Wei-Ning Lin
[081551@mail.fju.edu.tw](mailto:081551@mail.fju.edu.tw)

# Supplementary Figures and Tables

## Supplementary Figures

**(B)**

**(A)**


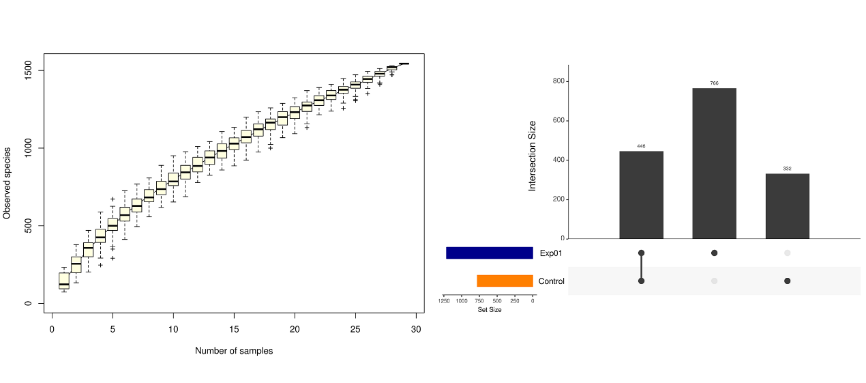


**Supplementary Figure 1.**

(A) Species accumulation curve. In the rank abundance curve, the x-axis presents the sequence numbers (sorted according to the number of ASVs), and the y-axis presents the relative abundance of the corresponding ASVs. Polylines of different colors represent the various samples. The horizontal axis represents the number of samples, and the vertical axis represents the number of ASVs. The results indicate the rate at which new species emerge with continual sampling. (B) UpSet plot. In the central block dot plot, each vertical row corresponds to an exclusive intersection (represented by solid circular points denoting the group names associated with that intersection). The top bar chart displays the intersection size, indicating the number of OTUs within each exclusive intersection. The bottom (left) bar chart displays the set size, indicating the number of OTUs within each group. Our study revealed that 766 ASVs were unique to patients with MG and that 332 ASVs were exclusive to individuals without MG. ASV, amplicon sequence variant; MG, myasthenia gravis; OUT, operational taxonomic unit.


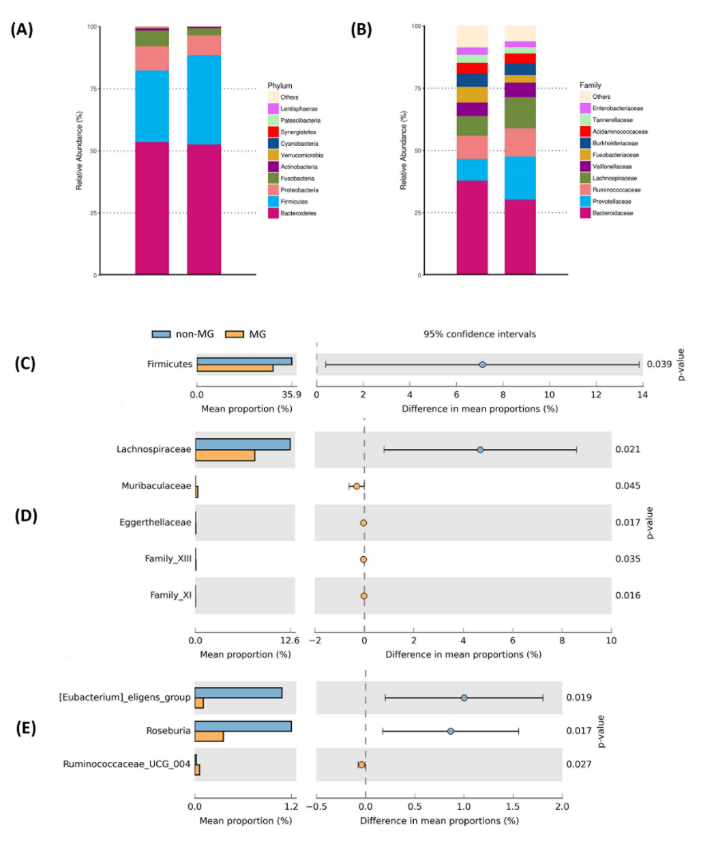


**Supplementary Figure 2.** Gut microbial compositions in the MG and non-MG groups.

The relative abundance levels of the dominant taxa at the (A) phylum level and (B) family level in each group are shown. The community bar plot shows the top 10 abundant gut microbes. To identify the taxa significantly varying between the MG and non-MG groups in terms of relative abundance, we performed Welch’s *t* test and statistically analyzed the metagenomic profiles. Microbial abundance levels at the (C) phylum, (D) family, and (E) genus levels in the MG (orange) and non-MG (blue) groups are shown. The bar plots represent the mean abundances; the between-group differences in mean abundance along with 95% confidence intervals are presented on the right. A *p* value of <0.05 was considered to be significant. The analysis at the phylum level indicated that Firmicutes were significantly depleted in the MG group (*p* < 0.05). The analysis at the family level indicated that the relative abundance of *Lachnospiraceae* sharply declined in the MG group (*p* < 0.05). The analysis at the genus level indicated that in the MG group, the relative abundance levels of *Roseburia* and *Eubacterium* sharply declined (*p* < 0.001), whereas that of *Ruminococcaceae* increased (*p* < 0.05). MG, myasthenia gravis.


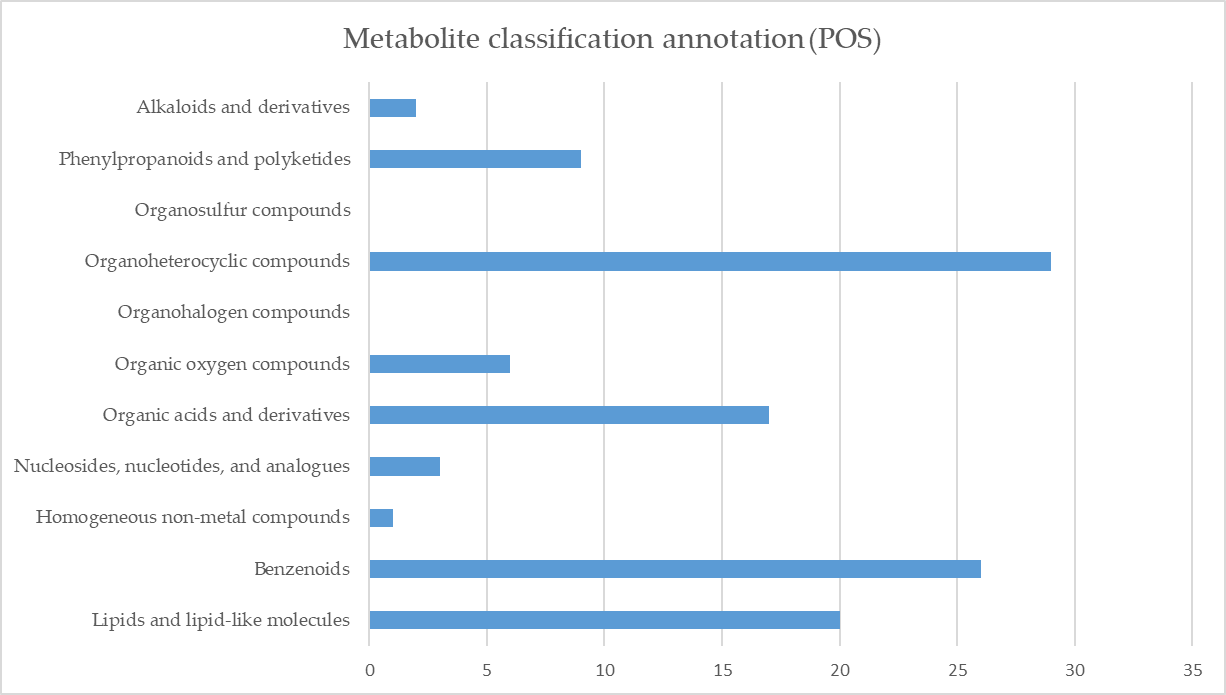


**Supplementary Figure 3.** Serum metabolite classification and annotation (at the superclass level) results obtained from the Human Metabolome Database: positive (POS) -ion mode.


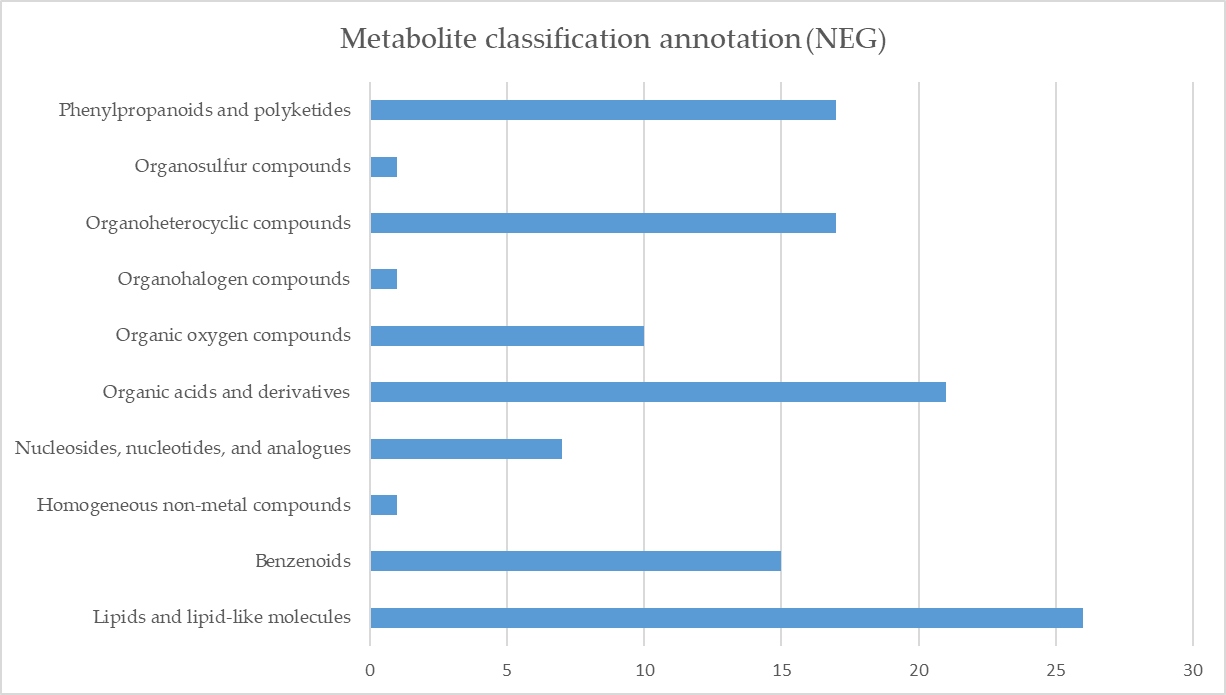


**Supplementary Figure 4.** Serum metabolite classification and annotation (at the superclass level) results obtained from the Human Metabolome Database: negative (NEG)-ion mode.


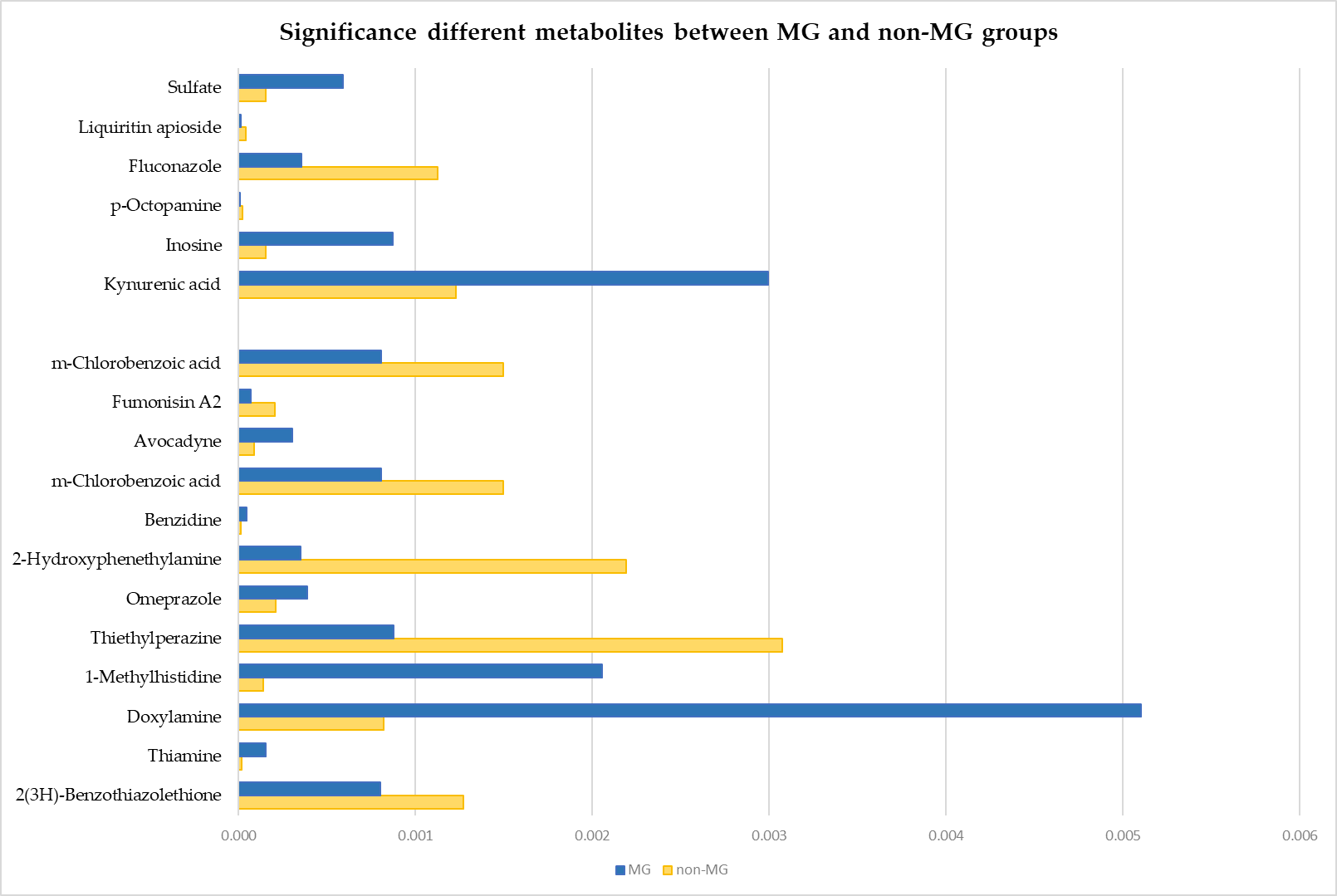


**Supplementary Figure 5.** Significant differences between the MG and non-MG groups in fecal metabolites. Blue indicates the MG group, and yellow indicates the non-MG group. MG, myasthenia gravis. *p* < 0.05, analysis of variance.

# Supplementary Appendix. Online Calculator for supportive diagnosis screening in Patients with Myasthenia gravis

An online calculator for supportive diagnosis screening for patients with MG based on gut microbiota-metabolites features (GutMGS). Available at <https://mgpredict.streamlit.app/>


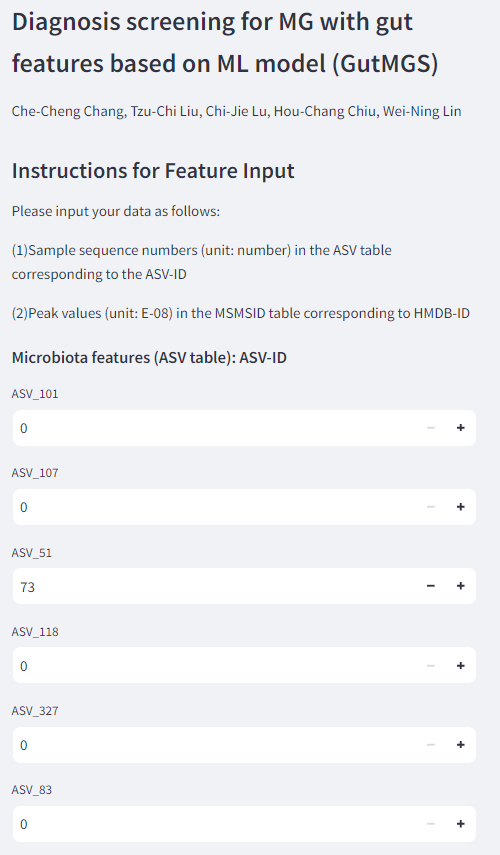

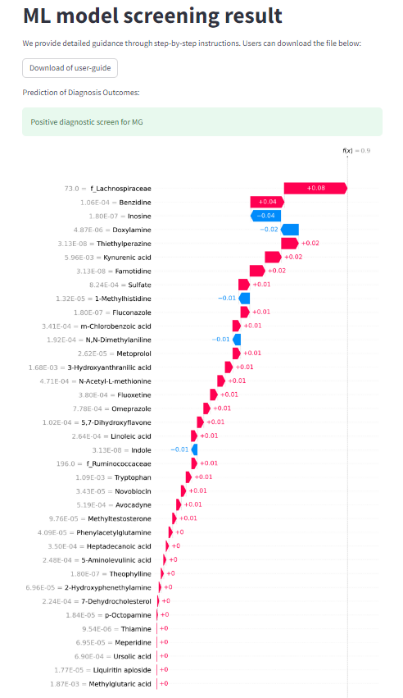


**Diagnosis screening for MG with gut features based on ML model (GutMGS)**

**Introduction:**

GutMGS is an online bioinformatics calculator designed for analysis of gut microbial-metabolite biomarkers for Myasthenia gravis (MG). GutMGS aims to simplify the standard metagenomics data analysis through SHapley Additive exPlanations (SHAP) analysis. The model is based on our ML model database from “Explainable machine learning model for identifying key gut microbes and metabolites biomarkers associated with myasthenia gravis”, which integrates data from 19 MG and 10 non-MG subjects. GutMGS offers users the capability to not only explore global explanations but also validate personalized explanations. Additionally, the platform provides data visualization tools for enhanced user readability. **Supplementary Figure 6** summarized the workflow of the online calculator


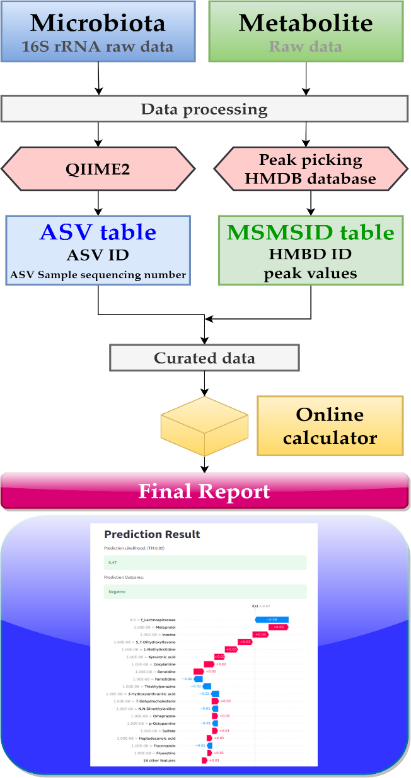


**Supplementary Figure 6.** The Summary workflow of online calculator (GutMGS)

**Data Input Instructions:**

Prior to utilizing the system and obtaining results, users are required to utilize the provided experimental software (QIIME2) and reference datasets (HMDB database) for data transformation. This is done to standardize the input IDs and variable values. After transforming the data using the aforementioned databases, users can input relevant numerical values.

Data Input: The first step is to input numerical values for the 48 features.: (i) microbiome valued: for 16s rRNA gene sequencing data, an ASVs abundance table are required based on QIIME2. (ii) Metabolome valued: a table with compound name, HMDB ID and chemical classification. Ensure that the sample IDs from microbiome and metabolome datasets are correctly matched with the respective spaces.

Upon obtaining the numerical outcomes, including: (1) Sample Sequence Numbers in ASV tables for each ASV-ID and (2) peak values (unit as 10^-8^ or E-08) in MSMSID stables for each HMDB-ID. Entering data in the corresponding fields facilitates the generation of SHAP results with and subsequent analysis for prediction screening.

表單的頂端

**Analysis of dataset and personalized measurement:**

Here is an illustrative example from our cross-sectional study conducted through 16S sequencing of the gut microbiome and UHPLC-MS/MS of gut metabolites, comparing MG subjects (MG) with healthy controls (non-MG). The ASV table, taxonomy name and sample sequence number is provided from QIIME 2. The Metabolites feature table (MSMSID table) and values is obtained from comparison with the HMDB datasets, including the HMDB-ID, compound name and peak values.


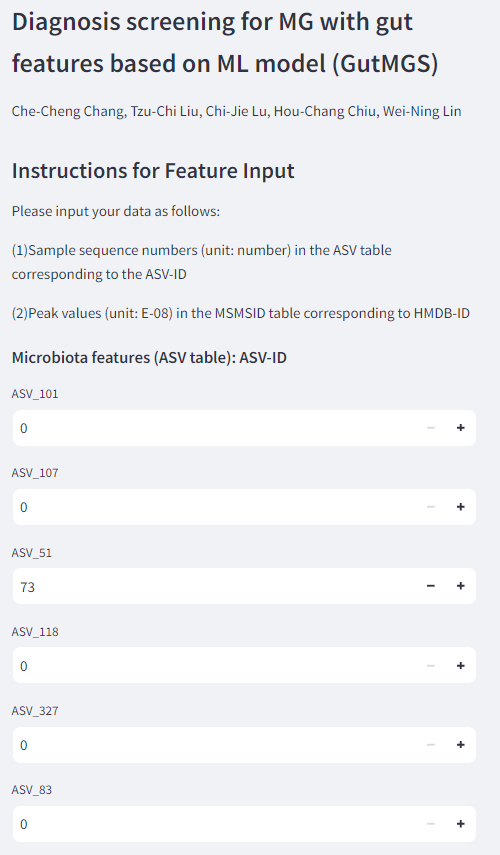

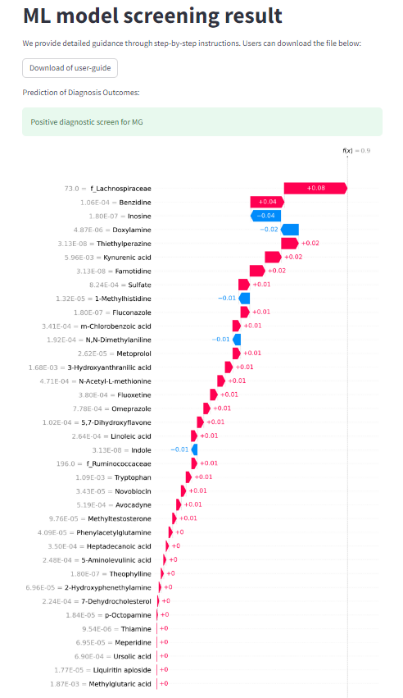


The prediction result is displayed in the colorful chart on the right side, which includes Prediction of Diagnosis Outcomes and automatically generated SHAP Waterfall plots for personalized local explanations. This integrated approach provides a comprehensive understanding of the potential biomarkers and with personalized explanation associated with MG.

**Detail user guidance**

We also provide detailed guidance through step-by-step instructions. Users can download the file by click the “Download of user-guide” button in the main page of the online calculator as illustrated in the following diagram:


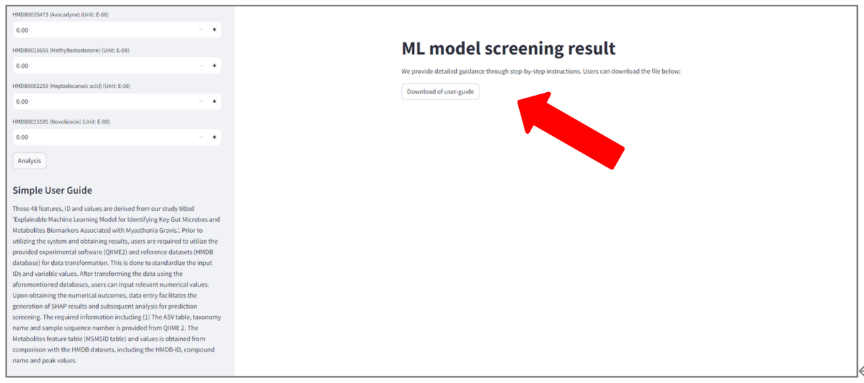


# Supplementary Tables

**Supplementary Table 1.** Characteristics of the MG and non-MG groups

| **Characteristics** | **MG** | **Non-MG** | ***P* value** |
| --- | --- | --- | --- |
| Sample size | 19 | 10 |  |
| Sex Female, *n* (%) | 13 (68) | 8 (80) | 0.8212 |
| Age (year) | 51.5 ± 14.4 | 49.8 ± 13.9 | 0.7731 |
| BMI (kg/m^2^) | 26.7 ± 4.4 | 24.3 ± 3.3 | 0.8141 |
| Comorbid disease, *n* (%) |  |  |  |
| Hypertension | 4 (21) | 2 (20) |  |
| Diabetes | 1 (5) | 1 (10) |  |
| Hyperlipidemia | 1 (5) | 2 (20) |  |
| Depression disorder | 1 (5) | 1 (10) |  |
| Hyperthyroidism | 2 (11) | 1 (10) |  |
| Disease duration (month) | 59.2 ± 77.8 | - | - |
| Medication |  |  |  |
| Pyridostigmine, *n* (%) | 19 (100) |  |  |
| Daily Pyridostigmine dose (mg) | 192 ± 114 |  |  |
| PSL, *n* (%) | 9 (47) |  |  |
| PSL dose per day (mg) | 9.2 ± 10.5 |  |  |
| Azathioprine, *n* (%) | 2 (11) |  |  |
| Serology of AChR antibody, *n* (%) | 18 (95) |  |  |
| Thymoma, *n* (%) | 8 (42) |  |  |
| PSL dose per day (mg) | 9.2 ± 10.5 |  |  |
| QMGS | 10.3 ± 4.2 | - | - |
| MGC | 8.5 ± 8.7 | - | - |
| Antibody titer (Nmol/L) | 81.2 ± 70.8 | - | - |

**Abbreviations:** AChR, acetylcholine receptor; BMI, body mass index; MG, myasthenia gravis; MGC, MG composite score; PSL, prednisolone; QMGS, Quantitative Myasthenia Gravis score; PSL, prednisolone.

**Supplementary Table 2.** Characteristics of the thymoma MG and non-thymoma MG groups

| **Characteristics** | **Thymoma MG** | **Non-thymoma MG** | ***p* value** |
| --- | --- | --- | --- |
| Sample size | 8 | 11 |  |
| Sex Female, *n* (%) | 6 (68) | 7 (64) | 1.000 |
| Age (year) | 45.9 ± 14.0 | 53.1± 16.1 | 0.152 |
| BMI (kg/m^2^) | 24.6 ± 3.1 | 24.5± 6.0 | 0.657 |
| Disease duration (month) | 67.5 ± 96.7 | 61.8± 77.8 | 1.000 |
| Medication |  |  |  |
| Pyridostigmine, *n* (%) | 8 (100) | 11 (100) |  |
| Daily Pyridostigmine dose (mg) | 202.5 ± 71.3 | 172.2 ± 130.0 | 0.904 |
| PSL, *n* (%) | 5 (62.5) | 3 (27.2) | 0.181 |
| PSL dose per day (mg) | 13.8 ± 12.5 | 6.9 ± 9.6 | 0.177 |
| Azathioprine, *n* (%) | 1 (12.5) | 1 (9.1) | 1.000 |
| QMGS | 7.5 ± 3.9 | 10.6 ± 1.8 | 0.016* |
| MGC | 4.0 ± 3.0 | 5.9 ± 2.3 | 0.033* |

**Abbreviations:** AChR, acetylcholine receptor; BMI, body mass index; MG, myasthenia gravis; MGC, MG composite score; PSL, prednisolone; QMGS, Quantitative Myasthenia Gravis score; PSL, prednisolone. **p*<0.05

**Supplementary Table 3.** Ranking results for 13 significantly contributing ASV (top 1%) features that were used to construct random forest models

| Ranking | ASV features | Importance score |
| --- | --- | --- |
| 1 | ASV_101 | 0.06 |
| 2 | ASV_107 | 0.052 |
| 3 | ASV_118 | 0.051 |
| 4 | ASV_327 | 0.045 |
| 5 | ASV_83 | 0.037 |
| 6 | ASV_51 | 0.027 |
| 7 | ASV_109 | 0.026 |
| 8 | ASV_131 | 0.014 |
| 9 | ASV_12 | 0.013 |
| 10 | ASV_593 | 0.008 |
| 11 | ASV_300 | 0.007 |
| 12 | ASV_255 | 0.004 |
| 13 | ASV_408 | 0.003 |

**Abbreviation:** ASV, amplicon sequence variant.

**Supplementary Table 4.** Ranking results for 35 significantly contributing metabolite features that were used to construct random forest models

| Ranking | Metabolite ID | Importance score | CpdName |
| --- | --- | --- | --- |
| 1 | FT1497 | 0.075 | Inosine |
| 2 | FT0255 | 0.047 | Kynurenic acid |
| 3 | FT1148 | 0.043 | Doxylamine |
| 4 | FT0388 | 0.035 | 1-Methylhistidine |
| 5 | FT0305 | 0.028 | Thiethylperazine |
| 6 | FT4134 | 0.025 | Famotidine |
| 7 | FT1291 | 0.023 | Metoprolol |
| 8 | FT0131 | 0.023 | 3-Hydroxyanthranilic acid |
| 9 | FT4119 | 0.021 | Sulfate |
| 10 | FT0646 | 0.02 | Indole |
| 11 | FT0766 | 0.02 | Fluconazole |
| 12 | FT2377 | 0.02 | Benzidine |
| 13 | FT2527 | 0.02 | Thiamine |
| 14 | FT0868 | 0.018 | Omeprazole |
| 15 | FT1439 | 0.017 | Liquiritin apioside |
| 16 | FT0374 | 0.016 | N,N-Dimethylaniline |
| 17 | FT0264 | 0.015 | Methylglutaric acid |
| 18 | FT0515 | 0.014 | p-Octopamine |
| 19 | FT1296 | 0.014 | 5,7-Dihydroxyflavone |
| 20 | FT0612 | 0.013 | N-Acetyl-L-methionine |
| 21 | FT0095 | 0.013 | m-Chlorobenzoic acid |
| 22 | FT0823 | 0.013 | Theophylline |
| 23 | FT4353 | 0.012 | 2-Hydroxyphenethylamine |
| 24 | FT5291 | 0.011 | 7-Dehydrocholesterol |
| 25 | FT3302 | 0.011 | Avocadyne |
| 26 | FT2821 | 0.01 | Linoleic acid |
| 27 | FT5579 | 0.009 | Phenylacetylglutamine |
| 28 | FT3383 | 0.009 | Fluoxetine |
| 29 | FT5647 | 0.009 | Ursolic acid |
| 30 | FT4140 | 0.009 | Heptadecanoic acid |
| 31 | FT7950 | 0.009 | Novobiocin |
| 32 | FT1032 | 0.008 | Meperidine |
| 33 | FT3314 | 0.008 | Methyltestosterone |
| 34 | FT0489 | 0.007 | 5-Aminolevulinic acid |
| 35 | FT0722 | 0.007 | Tryptophan |

**Supplementary Table 5.** Detailed description of the 13 significantly contributing ASV (top 1%) features that were used for model construction

| Ranking | ASV number | Phylum | Order | Family | Genus |
| --- | --- | --- | --- | --- | --- |
| 1 | **ASV_101** | Firmicutes | Clostridiales | *Lachnospiraceae* | *Eubacterium eligens group* |
| 2 | **ASV_107** | Firmicutes | Clostridiales | *Lachnospiraceae* | *Eubacterium eligens group* |
| 3 | **ASV_118** | Firmicutes | Clostridiales | *Lachnospiraceae* |  |
| 4 | **ASV_327** | Firmicutes | Clostridiales | *Lachnospiraceae* | *Lachnospiraceae UCG 010* |
| 5 | **ASV_83** | Firmicutes | Clostridiales | *Lachnospiraceae* | *Roseburia* |
| 6 | **ASV_51** | Firmicutes | Clostridiales | *Lachnospiraceae* |  |
| 7 | **ASV_109** | Firmicutes | Clostridiales | *Lachnospiraceae* | *Lachnoclostridium* |
| 8 | **ASV_131** | Firmicutes | Clostridiales | *Lachnospiraceae* | *Lachnospiraceae UCG 004* |
| 9 | **ASV_12** | Firmicutes | Clostridiales | *Ruminococcaceae* | *Faecalibacterium* |
| 10 | **ASV_593** | Firmicutes | Clostridiales | *Ruminococcaceae* | *Flavonifractor* |
| 11 | **ASV_300** | Firmicutes | Clostridiales | *Ruminococcaceae* | *Ruminococcaceae_UCG_002* |
| 12 | **ASV_255** | Firmicutes | Clostridiales | *Ruminococcaceae* | *Subdoligranulum* |
| 13 | **ASV_408** | Firmicutes | Clostridiales | *Lachnospiraceae* |  |

**Abbreviation:** ASV, amplicon sequence variant.
